# A Comparison between Triphenylmethyl and Triphenylsilyl Spirobifluorenyl Hosts: Synthesis, Photophysics and Performance in Phosphorescent Organic Light-Emitting Diodes

**DOI:** 10.3390/molecules28135241

**Published:** 2023-07-06

**Authors:** Wei Wei, Jie Ma, Jonas Schaab, Jason Brooks, Seogshin Kang, Matthew T. Whited, Peter I. Djurovich, Mark E. Thompson

**Affiliations:** 1 Department of Chemistry, University of Southern California, Los Angeles, CA 90089, USA; 2 Mork Family Department of Chemical Engineering and Materials Science, University of Southern California, Los Angeles, CA 90089, USA; 3 Universal Display Corporation, Ewing, NJ 08618, USA

**Keywords:** PHOLED, spirobiluorene, host morphology

## Abstract

This study presents the synthesis and characterization of two spirobifluorenyl derivatives substituted with either triphenylmethyl (**SB-C**) or triphenylsilyl (**SB-Si**) moieties for use as host materials in phosphorescent organic light-emitting diodes (PHOLED). Both molecules have similar high triplet energies and large energy gaps. Blue Ir(tpz)_3_ and green Ir(ppy)_3_ phosphorescent devices were fabricated using these materials as hosts. Surprisingly, **SB-Si** demonstrated superior charge-transporting ability compared to **SB-C**, despite having similar energies for their valence orbitals. In particular, **SB-Si** proved to be a highly effective host for both blue and green devices, resulting in maximum efficiencies of 12.6% for the Ir(tpz)_3_ device and 9.6% for the Ir(ppy)_3_ device. These results highlight the benefits of appending the triphenylsilyl moiety onto host materials and underscore the importance of considering the morphology of hosts in the design of efficient PHOLEDs.

## 1. Introduction

Phosphorescent organic light-emitting diodes (PHOLEDs) have been recognized as an efficient technology to achieve 100% internal efficiency in devices by harvesting both singlet and triplet excitons formed upon charge recombination [1,2,3]. Highly efficient and stable devices have been developed for green and red PHOLEDs. However, the performance of the blue PHOLEDs is still not comparable with their red and green counterparts, especially as the luminance efficiency for these devices degrades over long operational lifespans [4,5,6,7,8]. This limitation remains one of the most serious problems that needs to be addressed for widespread adoption of PHOLEDs in commercial applications.

The poor performance of blue PHOLEDs is related to properties of the host materials used in these devices. Compounds which include carbazolyl [9,10,11,12], phenylsilyl [13,14] and fluorenyl [15,16] derivatives have been widely used as hosts for PHOLEDs. However, these materials have several disadvantages in the blue PHOLEDs. Carbazolyl hosts such as (4,4′-*N*,*N*′-dicarbazole)biphenyl (**CBP**) and 4,4′,4″-tris(carbazol-9-yl)triphenylamine (**TCTA**) have been reported to be unstable in blue OLEDs [4,5,6]. Both Scholz et al. [4,5] and Kondakov at el. [6] found that the C-N bonds between the aryl and carbazolyl groups in these hosts dissociate during device operation and produce charged or radical species. Arylsilyl compounds have been considered as alternative host materials due to their strong Si-C bonds, and they have been utilized as large band gap host materials for blue PHOLEDs [13,14]. However, the arylsilyl derivatives have poor electrical conductivity, and their deep HOMO energies (>7.0 eV) inhibit hole injection from the hole transport layer into the emissive layer of the devices. Fluorenylsilanes, [15,16] which have both higher HOMO levels (6.6 eV) and charge mobilities, were developed later for use in blue PHOLEDs, but the large barrier for hole injection still leads to a low device efficiency.

Spirobifluorenyl derivatives have also been used as host materials in PHOLEDs, as they are reported to exhibit higher glass transition temperatures compared to the fluorenyl moieties [17]. Spirobifluorenyl species also have much higher hole mobilities than fluorenyl derivatives, although the latter have slightly higher electron mobilities [18]. As mentioned previously, the deep HOMO energy of the fluorenes (6.2–6.6 eV) could impede hole injection in blue OLEDs. Considering the similar HOMO levels of the spirobifluorenes, the greater hole carrier mobility of this species may improve the charge balance in the OLED devices. Several spirobifluorenyl-based hosts have been reported to achieve good thermal stability and avoid labile heteroatom linkages such as C-S, C-P or C-N by adding aromatic groups such as phenyl, triphenyl or spirobifluorenyl into the spirobifluorene backbone [17,19,20]. However, due to negligible steric hindrance between the moieties, these hosts have lower triplet energies compared to spirobifluorene (E_T_ = 2.8 eV), and therefore are poor candidates for confining triplet excitons on blue phosphorescent dopants in PHOLEDs. To address this issue, highly twisted spirobifluorenyl dimers and oligomers with an ortho–ortho linkage between spirobifluorenes moieties were introduced by Ma and Poriel et al., [21,22,23,24,25] which inhibited conjugation and retained the high triplet energy of the parent spirofluorene.

**Scheme 1:**
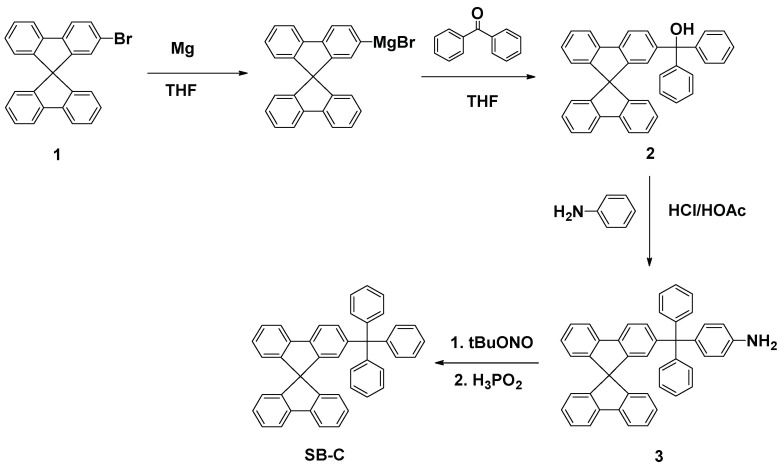
Synthesis of **SB-C**.

**Scheme 2:**
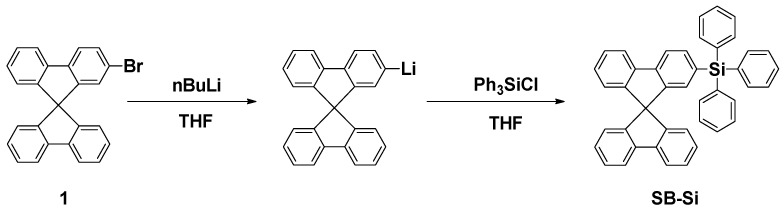
Synthesis of **SB-Si**.

In this study, a design strategy was utilized to maintain high triplet energy in spirobifluorenyl-based hosts, and two different derivatives were compared by introducing a tetravalent atom to isolate pendant phenyl rings. Specifically, a non-conjugated carbon core-based triphenylmethyl spirobifluorenyl compound, **SB-C** (Scheme 1), and its triphenylsilyl analog, **SB-Si** (Scheme 2), were synthesized. Various studies were conducted to compare the properties of these host materials, and their performance was evaluated in blue and green phosphorescent organic light-emitting diodes (PHOLEDs). Some of the results presented here appeared in one of the authors’ (Wei Wei) PhD thesis [26].

## 2. Results

### 2.1. Synthesis

Both of **SB-C** and **SB-Si** were designed to maintain high singlet and triplet energies using the pendant C or Si to isolate the aromatic groups of these two moieties. These two materials were synthesized using different synthetic methodologies starting from a common 2-bromo-9,9′-spirobifluorene (**1**) precursor. The synthesis of **SB-C** followed a four-step synthetic route, as shown in Scheme 1. A Grignard reagent prepared from **1** was treated with benzophenone to produce the diphenyl-9,9-spirobifluoren-2-yl methanol (**2**). A Friedel–Crafts reaction between **2** and aniline under a strong acidic condition afforded the *p*-(diphenyl-9,9-spirobifluoren-2-yl)methyl aniline (**3**). The amino group of **3** was then removed using tBuONO and H_3_PO_2_ to yield the final product (**SB-C**). The overall yield of this multi-step synthesis was 33%. **SB-Si** was synthesized more efficiently, as shown in Scheme 2. The addition of Ph_3_SiCl to 2-lithio-9,9′-spirobifluorene results in the **SB-Si** with a yield of 78% as a pure isolated product [24,25].

### 2.2. X-ray Structures

**Figure 1:**
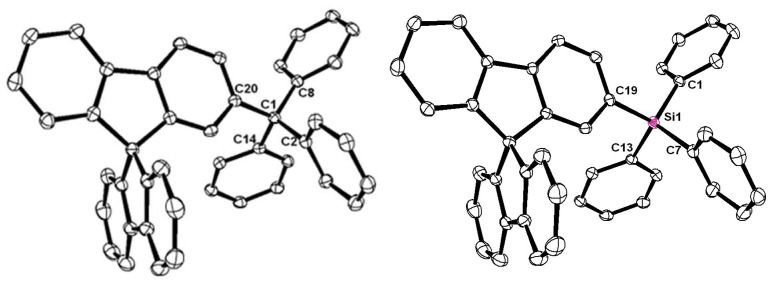
X-ray crystal structures of **SB-C** (**left**) and **SB-Si** (**right**).

X-ray crystal structures for **SB-C** and **SB-Si** were obtained and are shown in Figure 1. The bond distance between the spirobifluorenyl carbon and the triphenylmethyl carbon center in **SB-C** (C1-C20 = 1.547 Å) is shorter than for the equivalent bond in **SB-Si** (Si1-C19 = 1.873 Å), as are the bond distances between the sp^3^ center to the atoms on the phenyl rings (C1-C_Ph_ = 1.548–1.554 Å for **SB-C** and Si1-C_Ph_ = 1.874–1.880 Å for **SB-Si**). The packing of the molecules in a unit cell of each of the materials is shown in Figure S1. No overlap of the aromatic rings was found for **SB-C**, whereas a partial overlap was observed in **SB-Si**. However, it is worth noting that the shortest full face-to-face distance in **SB-Si** was approximately 5 Å, which is too long to be effective for π-π stacking interactions.

### 2.3. Theoretical Calculations

**Figure 2:**
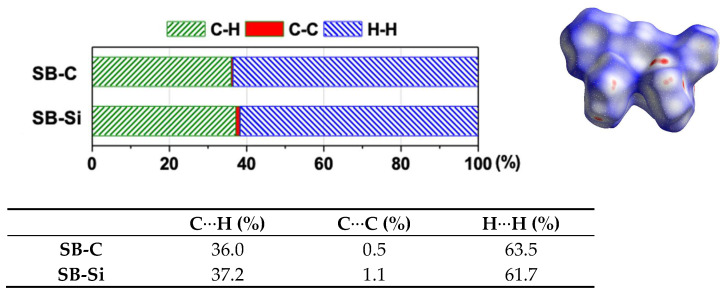
Intermolecular interactions between molecules in **SB-C** and **SB-Si** crystals calculated using Crystal Explorer software package. Different colors indicate different types of close intermolecular interactions (C···H, C···C or H···H). The *x*-axis shows the percentage of each interaction and is tabulated below. An illustration of a Hirshfeld surface for **SB-C** is shown on the right.

In order to quantify other intermolecular interactions, Hirshfeld surface calculations were applied in conjugation with the X-ray data to explore the interatomic distances of these two materials. A Hirshfeld surface identifies regions where the intermolecular interactions of a molecule in the crystal lattice are shorter than a boundary defined by a hypothetical van der Waals surface calculated for the isolated molecule [27]. Areas of the molecules where the intermolecular interactions are closer can be quantified and compared to other regions where the interactions are longer (Figure S2). Then, the fingerprint generated from the Hirshfeld surface can provide detailed information regarding the types and percentages of these interactions. Crystalline **SB-Si** showed higher ratios of both C···C and C···H type interactions between molecules than found in **SB-C**, which instead had a higher ratio of H···H type intermolecular interactions (Figure 2). Density functional theory (DFT) calculations also determined the energies for the highest occupied and lowest unoccupied molecular orbitals (HOMO and LUMO) of the two molecules (Figure S3). These two molecules have similar HOMO and LUMO surfaces. The HOMO is delocalized across the entire spirobifluorenyl moiety, whereas the LUMO is localized on the fluorenyl linkage substituted to the Ph_3_C- or Ph_3_Si- groups. The calculated HOMO and LUMO levels for both molecules were −5.6 eV and −1.0 eV, respectively.

### 2.4. Electrochemistry

The electrochemical properties of **SB-C** and **SB-Si** were examined using cyclic voltammetry (CV) and differential pulse voltammetry (DPV) (Table 1 and Figure S4). The electrochemical reversibility was established using CV measurements, whereas the redox potentials were determined using DPV and referenced to an internal ferrocenium/ferrocene couple. Both compounds are oxidized irreversibly at ca. 1.3 V. Similar irreversible oxidation potentials have been reported for the parent spirobifluorene and its non-conjugated derivatives [28,29]. A second irreversible oxidation wave was also observed at 1.7 V for both **SB-C** and **SB-Si** (Figure S4a). These two waves are similar to oxidative processes that have been reported for other spirobifluorenes [28,29]. The first oxidation wave was thus assigned to oxidation of the fluorenyl moiety bound to the CPh_3_ or SiPh_3_ group, whereas the second wave was assigned to oxidation and polymerization of the unsubstituted fluorenyl moiety. The reduction waves for **SB-C** and **SB-Si** are also irreversible and were detected near the edge of the solvent window at −3.0 V for **SB-C** and −2.8 V for **SB-Si** (Table 1, Figure S4a). Energy levels for the HOMO and LUMO were estimated from the electrochemical data of these two molecules using correlations between the redox potentials. The HOMO levels were −6.2 and −6.3 eV for **SB-C** and **SB-Si**, respectively, whereas the LUMO levels were −1.3 eV for the **SB-C** and −1.5 eV for the **SB-Si**. The HOMO energies are similar to the value reported for **SB-Si** (−6.2 eV) obtained using photoemission yield spectroscopy [25]. Moreover, the oxidation potential observed for **SB-Si** (1.34 V) is close to values reported for a series of silicon-substituted dimethylfluorenyl compounds (*E*_ox_ = 1.4 V) [16]. The transport gaps calculated from the difference of the HOMO and LUMO levels are 4.9 eV and 4.8 eV for **SB-C** and **SB-Si**, respectively. Hence, the large energy gaps of **SB-C** and **SB-Si** are comparable to their fluorenyl counterparts, which make them promising candidates for hosting blue dopants.

### 2.5. Thermal Properties

The glass transition temperatures (T_g_) of the materials are considered to be an important factor for obtaining homogenous thin films in OLED devices [31,32,33,34]. Due to the steric bulk of the spirobifluorenyl substituent, a high glass transition temperature of 87 °C was observed for **SB-Si** (Table 2 and Figure S5). In contrast, we were not able to detect the glass transition temperature of **SB-C**. The melting temperature of **SB-C** (T_m_ = 284 °C) is also higher than that of **SB-Si** (T_m_ = 253 °C) (Table 2 and Figure S5). The sublimation temperatures of these two molecules are not influenced by either the C or the Si center, and both compounds can be sublimed at 245 °C in high yield (80–90%) using a gradient vacuum sublimator. This makes these two molecules suitable for OLED device fabrication using vapor deposition methods.

### 2.6. Photophysics

**Figure 3:**
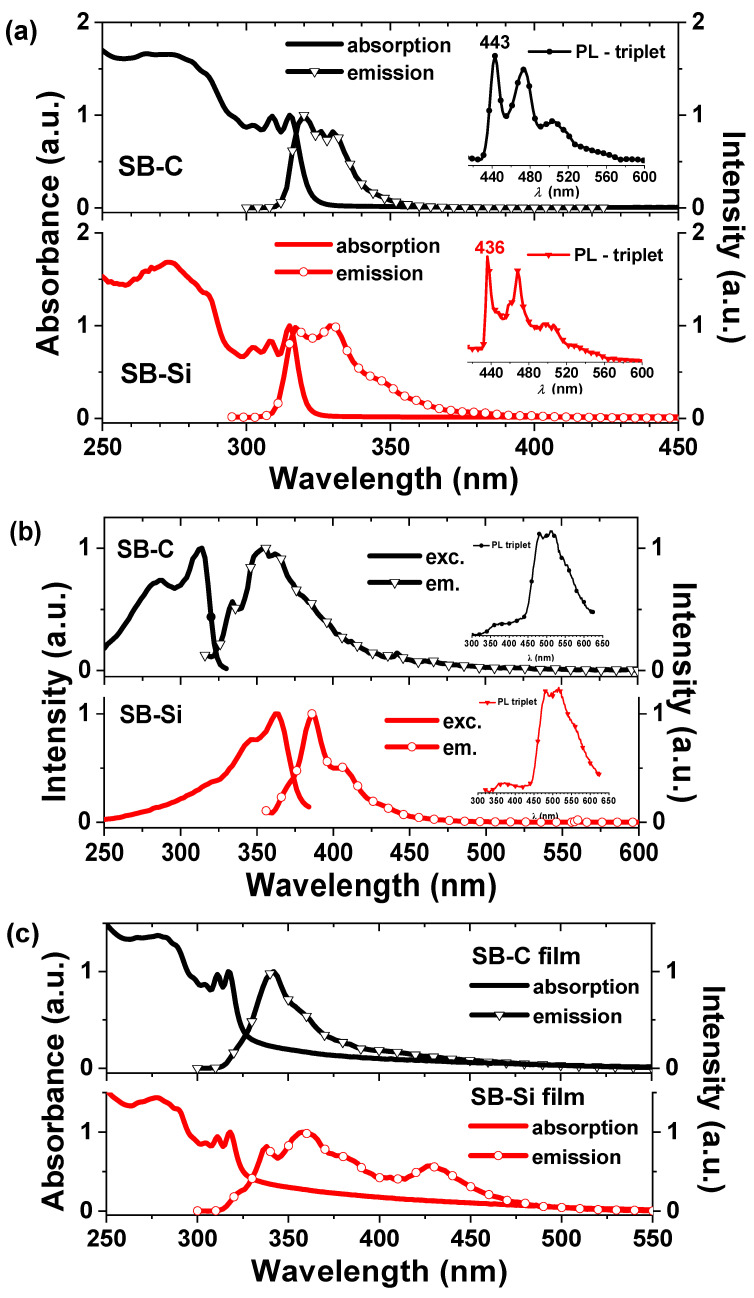
(**a**) Absorption and emission spectra of **SB-C** and **SB-Si** in 2-MeTHF solution. (**b**) Excitation and emission spectra of **SB-C** and **SB-Si** from sublimed crystals. (**c**) Absorption and photoluminescence (PL) spectra of **SB-C** and **SB-Si** from amorphous thin films (40 nm).

The UV–visible absorption and photoluminescence (PL) spectra of **SB-C** and **SB-Si** in 2-methyltetrahydrofuran (2-MeTHF) solutions are shown in Figure 3a. The absorption bands appearing between 290 nm to 320 nm were attributed to π-π transitions on the spirobifluorene [35,36]. The lowest energy transitions of both molecules were at 315 nm (Table 3). The optical gaps determined from the long wavelength edge of the absorption spectra were near identical for the **SB-C** (*λ* = 317 nm, 3.9 eV) and the **SB-Si** (*λ* = 316 nm, 3.9 eV). These optical gaps were ~1 eV smaller than the transport gaps determined from their respective redox potentials. Singlet emission typical for the fluorenyl moiety was observed from **SB-C** (*λ*_max_ = 320 nm) and **SB-Si** (*λ*_max_ = 318 nm) (Table 3 and Figure 3a). The phosphorescence spectra of **SB-C** (*λ*_max_ = 443 nm) and **SB-Si** (*λ*_max_ = 438 nm) were observed at low temperature (77 K gated), as shown in Table 3 and Figure 3a (inset). The triplet energies for both molecules (E_T_ = 2.80 eV) are only slightly lower compared to the parent fluorenylsilanes (*λ*_max_ = 435 nm, E_T_ = 2.85 eV) [16].

The excitation and emission spectra of crystals of **SB-C** and **SB-Si** were also measured so that a direct correlation with their molecular arrangements in their solid state could be made using the X-ray and photophysical data (Figure 3b). The excitation spectrum of **SB-C** crystals shows a maximum peak at 314 nm, which is 3 nm blue-shifted from its solution absorption. A 36 nm red shift was also observed for the emission spectrum of crystalline **SB-C** from its spectrum in solution. In contrast, the excitation spectrum of **SB-Si** crystals shows a strong red shift (47 nm) from to its solution absorption, giving a maximum excitation at 362 nm (Table 3, Figure 3b). Associated with the red shift of the excitation, the emission of this compound is also red shifted for 59 nm from its emission in solution. The red-shifted emission spectra of **SB-C** and **SB-Si** come from the intermolecular interactions between molecules in the crystals. The large red shifts of both the excitation and emission spectra of **SB-Si** suggest that a greater number of close intermolecular interactions are present in the crystals than in **SB-C**. The Hirshfeld surface calculation discussed in the previous section also provided evidence that stronger intermolecular, C···C type interactions are present in the **SB-Si** crystals. However, similar phosphorescence spectra for **SB-C** and **SB-Si** (*λ*_max_ = 450 nm) were observed at 77 K for both compounds (Table 3, Figure 3b inset).

It is particularly important to investigate the photophysics of **SB-C** and **SB-Si** in amorphous thin films, since these two materials were designed for thin-film semiconductor devices. Therefore, the absorption and emission spectra of the vacuum-deposited thin films were measured and are shown in Figure 3c. The lowest energy absorption transitions appear at 318 nm for both materials and are close to values measured in dilute solution. The gradually sloping absorption band after 330 nm is due to light scattering. Even though films of these two compounds have similar absorption spectra, their emission spectra are quite different. Emission from the **SB-C** film peaks at 342 nm, whereas two different, red-shifted bands in **SB-Si** peak at 360 nm and 430 nm. The bands between 330 and 400 nm are assigned to be singlet emission from the molecules that are red shifted in the solid state. The red shift could be due to weak interactions between molecules in the films, considering that there are no interactions between individual molecules in a dilute solution. The emission band at 430 nm in **SB-Si** is similar to the emissions observed from solid films of dimethylfluorenyl silanes [16], and has been assigned to fluorescence from more strongly aggregated molecules which act as luminescent trap states.

### 2.7. OLED Studies

**Figure 4:**
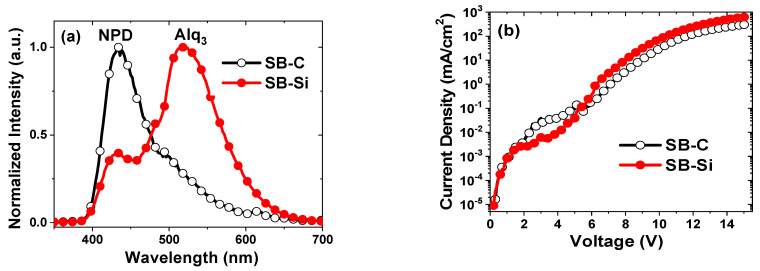
(**a**) EL spectra at 9 V and (**b**) *J-V* plot of undoped **SB-C** and **SB-Si** devices.

The charge transport properties of **SB-C** and **SB-Si** were evaluated by comparing the performance of undoped OLED devices fabricated with the structure ITO/NPD (30 nm)/mCP (10 nm)/**SB-C** or -Si host (20 nm)/Alq_3_ (20 nm)/LiF (1 nm)/Al (100 nm) (Figure S6). The electroluminescence (EL) spectra of each host were voltage-dependent in the range 8 V–15 V for both **SB-C** and **SB-Si** (Figure S6). With increasing voltage, emission from NPD increases whereas emission from Alq_3_ decreases. However, the **SB-C** device yields predominant emission from NPD in the whole range of examined potentials, whereas the **SB-Si** device displays predominant emissions from Alq_3_ (Figure 4a). Since these two materials have similar HOMO and LUMO energies, the devices should have a similar charge injection barrier for holes and electrons. Thus, the different EL spectra of these two materials may come about from the difference in the carrier mobilities in the two materials. The current voltage (*J-V*) characteristics of these two devices are shown in Figure 4b. A larger current was observed for the **SB-Si** device at high voltage. This difference could be because the **SB-Si** film has a higher percentage of aromatic rings in close enough proximity to facilitate charge transfer than does **SB-C**. This is consistent with the Hirshfeld surface calculations, which suggest that **SB-Si** has a larger proportion of C···C intermolecular interactions in the crystalline state than **SB-C**.

**Figure 5:**
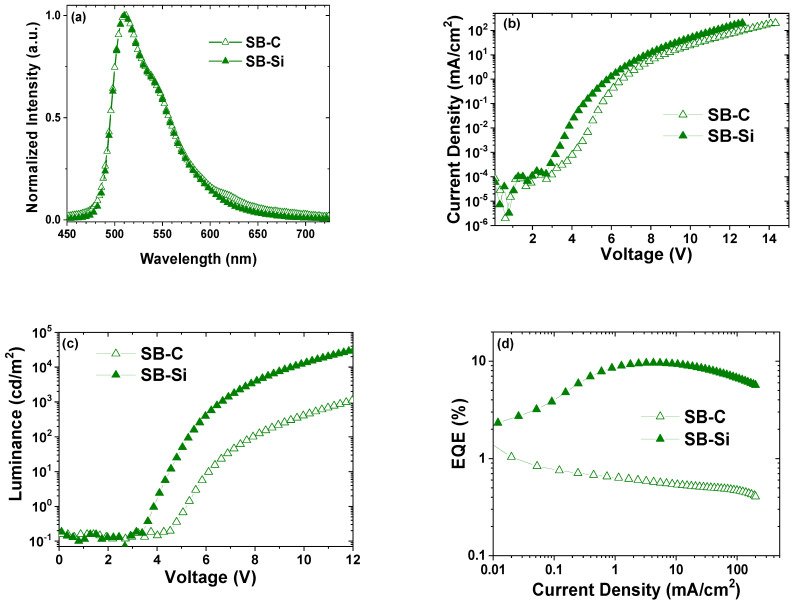
OLED device characteristics of Ir(ppy)_3_ devices. (**a**) EL spectra, (**b**) *J-V*, (**c**) *L-V* and (**d**) EQE–current density plots.

Phosphorescent devices using the green emissive dopant tris(2-phenylpyridyl)iridium(III) Ir(ppy)_3_ were fabricated to investigate the properties of two hosts in PHOLEDs. The device structure chosen for these devices was ITO/HATCN (10 nm)/NPD (30 nm)/(9% Ir(ppy)_3_: **SB-C** or -Si host (30 nm)/BAlq (10 nm)/Alq_3_ (40 nm)/LiF (1 nm)/Al (100 nm), using hexaazatriphenylenehexacarbonitrile (**HATCN**) as a hole injection material and bis(2-methyl-8-quinolinolato-N,O)-(1,1′-biphenyl-4-olato)aluminum (**BAlq**) as a hole-blocking material. It was necessary to dope Ir(ppy)_3_ at a high level in these devices, as the dopant must carry both holes and electrons due to the wide energy band gap of the host. The performance of these two devices is summarized in Table 4. The EL spectra of these two devices are shown in Figure 5a. Both devices achieved good charge balance at the emissive layer, leading to pure Ir(ppy)_3_ emission at 511 nm. The current density–voltage–luminance (*J-L-V*) characteristics of these two devices are shown in Figure 5b,c. A higher current density and brightness was found at all voltages for the **SB-Si** device than for the **SB-C** device. Correspondingly, the **SB-Si** device turns on at a lower voltage (*V*_turn-on_ = 3.9 V) than the **SB-C** device (*V*_turn-on_ = 5.2 V). The device with the **SB-Si** host shows a maximum external quantum efficiency (EQE) of 9.6% at a current density of 4.24 mA/cm^2^, whereas the maximum EQE of the **SB-C** device is only 1.2% (Figure 5d). Ir(ppy)_3_ is prone to aggregation at these high doping concentrations, which leads to lower efficiency for the devices. Therefore, these Ir(ppy)_3_ devices provide a good measure of the ability of the host material to disperse the dopant. The high performance of the **SB-Si** device shows that this material is significantly better at dispersing Ir(ppy)_3_ than **SB-C**. The EQE obtained using **SB-Si** also agrees with data from previously published OLED devices using Ir-based emitters in the same **SB-Si** host [24,25].

**Figure 6:**
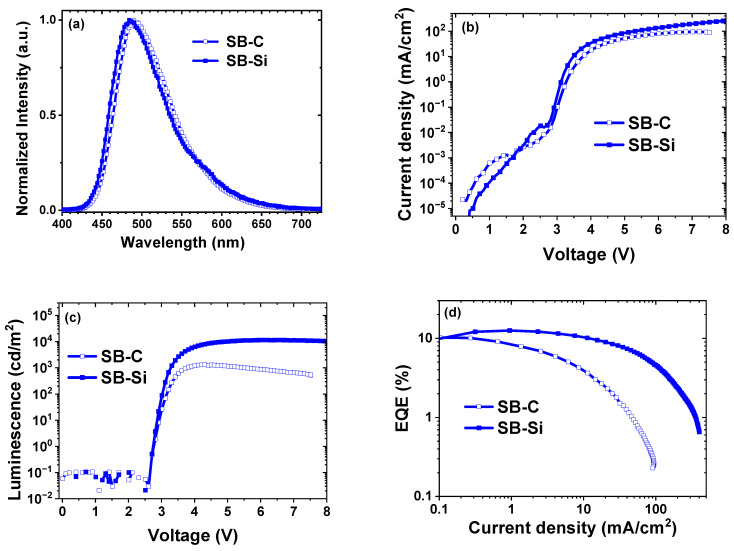
OLED device characteristics of Ir(tpz)_3_ devices. (**a**) EL spectra, (**b**) *J-V*, (**c**) *L-V* and (**d**) EQE–current density plots.

The two host materials were also examined in blue PHOLED devices using tris(*N*,*N*-di-*p*-tolyl-pyrazinoimidazol-2-yl)iridium(III) (Ir(tpz)_3_) as the phosphorescent emitter [37]. A doping weight percentage of 20% was determined to be optimal for these devices and host materials, as other concentrations showed a worse performance. To see the difference in a sky-blue material, which is not as prone to aggregation as Ir(ppy)_3_, and to compare **SB-Si** with **SB-C**, the following device structure was used: ITO/HATCN (5 nm)/TAPC (40 nm)/**SB-C** or -Si (10 nm)/(20% Ir(tpz)_3_: **SB-C** or -Si host, 25 nm)/BP4mPY (60 nm)/LiF (1 nm)/Al (100 nm). The performance of these two devices is summarized in Table 4. Here, di-[4-(*N*,*N*-di-*p*-tolyl-amino)-phenyl]cyclohexane (**TAPC**) was used as a hole-transporting layer, 3,3′,5,5′-tetra[(m-pyridyl)-phen-3-yl]biphenyl (**BP4mPY**) was used as an electron-transporting layer, and 10 nm undoped host was used as an electron-blocking layer. Electroluminescence only from Ir(tpz)_3_ was observed for both the **SB-C** and **SB-Si** devices (Figure 6a). The **SB-Si** device exhibited higher current conduction and luminance than the **SB-C** device at any given voltage (Figure 6b,c). For example, at 4 V the **SB-C** device had a current density of 17.8 mA/cm^2^ and a luminance of 1200 cd/m^2^, whereas the **SB-Si** device reached a current that was two times larger (35 mA/cm^2^) and a luminance that was five times higher (6300 cd/m^2^). The **SB-C** device reached its highest efficiency (EQE = 10.2%) with a current density of 0.01 mA/cm^2^ (Figure 6d). A significant decrease in the external efficiency was observed in the **SB-C** device starting from a current density of 0.7 mA/cm^2^, which could be due to triplet–triplet annihilation. The **SB-Si** device reached its maximum external quantum efficiency (12.6%) at a current density of 1 mA/cm^2^, and no significant drop in EQE was observed for this device until the current density increased to 10 mA/cm^2^ (Figure 6d). Overall, devices using the **SB-Si** host exhibited superior conductivity, higher luminance and enhanced EQE at any given voltage over devices using the **SB-C** host. These improvements can be attributed to the increased intermolecular C···C interactions in **SB-Si**, as suggested by the calculated and photophysical data.

## 3. Materials and Methods

### 3.1. Synthesis

All chemicals and solvents were purchased from commercial sources without further purification except for tetrahydrofuran (THF), which was distilled over sodium/benzophenone.

*Triphenyl-9,9-spirobifluoren-2-yl silane* (**SB-Si**) [24,25]. To a solution of 0.78 g (2 mmol) 2-bromo-9,9-spirobifluorene (**1**) in dry THF was added 1.38 mL (2.2 mmol) of n-BuLi (1.6 M in hexanes) under an N_2_ atmosphere to obtain an orange solution. Triphenylchlorosilane (0.56 g, 1.9 mmol) was initially weighed and dissolved in THF under an N_2_ atmosphere and was added using a syringe into the solution after 1 h. The solution was stirred overnight. Ethyl acetate and water were used to extract the organic layer, which was later dried over brine and magnesium sulfate. Organic solvents were reduced by rotary evaporation to obtain an orange-colored solid that was recrystallized in CH_2_Cl_2_ to obtain 0.89 g of a white solid product.

*Triphenyl-9,9-spirobifluoren-2-yl methane* (**SB-C**). A solution of 1.74 g **1** was added dropwise to Mg in 8 mL of THF. A solution of benzylphenone in 4 mL of THF was slowly added into the prepared Grignard reagent at room temperature, and the mixture was refluxed for three hours. After being cooled down to room temperature, ammonium chloride was used to quench the reaction. The organic layer was extracted by CH_2_Cl_2_ and dried over MgSO_4_. After the solvent was removed using rotary evaporation, a silica gel column with CH_2_Cl_2_/hexanes (1:3) was performed to yield 1.63 g of diphenyl-spirobifluorenyl methanol (**2**) as a white solid a yield of 81%. Compound **2** (3.17 mmol, 1.58 g) was refluxed with aniline (4.8 mmol, 0.44 mL), HCl (0.5 mL) and acetic acid (10 mL) at 140 °C for 3 days. The reaction mixture was allowed to cool down to room temperature, and 100 mL of water was added. NaOH solid was added until the solution became neutral. The product was filtered out as 1.65 g of a white solid of diphenylspirobifluorenylaniline methane (**3**). To a solution of 0.57 g of **3** in 30 mL of THF, a solution of 6 mmol of H_3_PO_2_ in H_2_O (0.5 mL) was added. After stirring for 10 min, a solution of *t*BuONO in 5 mL of THF was added, and the mixture was stirred for 16 h at 40 °C. After the THF was removed, the reaction mixture was extracted with CH_2_Cl_2_ and water. The organic layer was washed with brine and dried over MgSO_4_. After the solvent was removed with reduced pressure, recrystallization was performed to yield 2.51 g of a white solid as the product.

The ^1^H NMR, mass spectrometry and elemental analysis data of these compounds are shown as follows:

*Triphenyl-9,9-spirobifluoren-2-yl silane* (**SB-Si**), yield 78%. ^1^H NMR (CDCl_3_, 250 Hz): δ 7.80–7.87 (2H, dd, Ar-H), 7.74–7.78 (2H, d, Ar-H), 7.45–7.49 (1H, d, Ar-H), 7.29–7.41 (12H, m, Ar-H), 7.20–7.27 (5H, m, Ar-H), 7.08–7.15 (4H, dd, Ar-H), 7.05 (1H, s, Ar-H), 6.75–6.79 (2H, d, Ar-H), 6.71–6.75 (1H, d, Ar-H). MS (*m*/*z*): 574, 497, 316, 259. Ana. Calcd. for C_43_H_30_Si: C 89.85 H 5.26; found: C 90.07 H 5.12.

*Diphenyl-9,9-spirobifluoren-2-yl methanol* (**2**), yield 81%. ^1^H NMR (CDCl_3_, 400 Hz) δ: 7.79–7.81 (1H, d, Ar-H), 7.77–7.79 (2H, d, Ar-H), 7.69–7.71 (1H, d, Ar-H), 7.33–7.38 (1H, d, Ar-H), 7.31–7.36 (2H, dd, Ar-H), 7.18–7.22 (4H, d, Ar-H), 7.18–7.21 (2H, dd, Ar-H), 7.12–7.17 (4H, dd, Ar-H), 7.04–7.10 (1H, dd, Ar-H), 6.92 (1H, s, Ar-H), 6.72–6.75 (2H, d, Ar-H), 6.67–6.70 (1H, d, Ar-H). MS (*m*/*z*): 498, 482, 421, 315.

*p-(Diphenyl-9,9-spirobifluoren-2-yl)methyl aniline* (**3**), yield 91%. ^1^H NMR (CDCl_3_, 250 Hz): δ 7.75–7.79 (1H, d, Ar-H) 7.69–7.73 (2H, d, Ar-H), 7.25–7.32 (3H, m, Ar-H), 7.21–7.24 (1H, d, Ar-H), 7.01–7.10 (11H, m, Ar-H), 6.80–6.83 (2H, d, Ar-H), 6.68–6.71 (2H, d, Ar-H), 6.65–6.68 (1H, d, Ar-H), 6.56–6.58 (1H, s, Ar-H), 6.40–6.43 (2H, d, Ar-H). MS (*m*/*z*): 572, 481, 258.

*Triphenyl-9,9-spirobifluoren-2-yl methane* (**SB-C**), yield 45%. ^1^H NMR (CDCl_3_, 400 Hz) δ: 7.76–7.79 (1H, d, Ar-H), 7.71–7.73 (2H, dd, Ar-H), 7.68–7.70 (1H, d, Ar-H), 7.30–7.34 (1H, dd, Ar-H), 7.27–7.31 (2H, dd, Ar-H), 7.23–7.26 (1H, d, Ar-H), 7.03–7.12 (18H, m, Ar-H), 6.68–6.70 (2H, d, Ar-H), 6.62–6.68 (1H, d, Ar-H), 6.55 (1H, s, Ar-H). MS (*m*/*z*): 558, 481, 403, 316. Ana. Calcd. for C_44_H_30_: C 94.59 H 5.41; found: C 94.45; H 5.31.

### 3.2. X-ray Crystallography

X-ray-quality crystals were grown as indicated in the experimental procedures for each complex, and the crystals were mounted on a nylon fiber with Paratone-N oil. All crystals were measured at 100 K with a Rigaku Xta LAB Synergy S, equipped with an HyPix-600HE detector and an Oxford Cryostream 800 low temperature unit, using a Cu Kα PhotonJet-S X-ray source. The frames were integrated using the SAINT algorithm to give the hkl files. The data were corrected for absorption effects using the multi-scan method (SADABS) with Rigaku CrysalisPro. The structures were solved by intrinsic phasing and refined with the SHELXTL software package.4. Structures of **SB-C** and **SB-Si** were deposited in the CCDC (2221201 and 2216982, respectively).

### 3.3. Thermal Analysis

Differential scanning calorimetry (DSC) was performed on a TA Instruments DSC Q10 instrument with a scanning range from room temperature to 300 °C. The sample was first scanned at a heating rate of 10 °C min^−1^ and was cooled down to room temperature rapidly using liquid N_2_. The second and third scans were performed at a heating rate of 5 °C min^−1^. The glass transition temperatures were determined from either the second or the third scan for each compound.

### 3.4. Electrochemistry and Photochemistry

Cyclic voltammetry was performed using an EG&G potentiostat/galvanostat model 283 under an N_2_ atmosphere. A glass carbon rod was used as the working electrode. Tetrabutylammonium hexafluorophosphate (TBAPF_6_, 0.1 M) was used as the supporting electrolyte. Anhydrous acetonitrile was used as the solvent for the oxidation measurements, and anhydrous DMF was used as the solvent for the reduction measurements. The redox potentials were calculated relative to an internal reference ferrocenium/ferrocene (Cp_2_Fe^+^/Cp_2_Fe). The UV–visible absorption spectra were measured by a Hewlett-Packard 4853 diode array spectrophotometer. The singlet and triplet emission measurements were performed on a Photon Technology International QuantaMaster model C-60SE spectrofluorimeter at room temperature and 77 K.

### 3.5. Device Fabrication

The OLED devices were fabricated on pre-patterned ITO-coated glass substrates (20 ± 5 Ω-cm^2^, Thin Film Devices, Inc., Anaheim, CA, USA). Prior to deposition, the substrates were cleaned with soap, rinsed with deionized water and sonicated for 10 min. Afterwards, two subsequent rinses and 15-min sonication baths were performed in acetone and isopropyl alcohol sequentially, followed by 10 min of UV ozone exposure. EVO Vac 800 deposition system from Angstrom Engineering, at ~6 × 10^−7^ Torr. current–voltage–luminescence (*J*-*V*-*L*) curves were measured in an inert atmosphere using a Keithley power source meter model 2400 and a Newport multifunction optical model 1835-C with PIN-220DP/SB blue-enhanced silicon photodiodes (OSI Optoelectronics Ltd., Hawthorne, CA, USA). The electroluminescence (EL) spectra of OLEDs were measured using the fluorimeter (Photon Technology International QuantaMaster model C-60SE) at several different voltages. The thicknesses were determined on silicon wafers using a Filmsense FS-1 ellipsometer.

## 4. Summary

In this research, two spirobifluorenyl compounds, **SB-C** and **SB-Si**, were designed as host materials and analyzed for their chemical composition, optoelectronic properties, and performance in OLED devices. Both compounds have large HOMO/LUMO gaps (4.7 ± 0.2 eV) and high triplet energies (2.8 eV). Despite their similarities, Hirshfeld surface calculations and photophysical analysis of the compounds in solid-state crystals revealed that **SB-Si** displays more intermolecular C···C interactions than does **SB-C**. In both undoped devices and PHOLED devices, the **SB-Si** material demonstrated superior charge-transporting abilities compared to the **SB-C**, as indicated by better current conduction and higher brightness. These findings illustrate the relationship between the material morphology and properties, and their impact on charge transport in PHOLED devices. Furthermore, while triplet–triplet annihilation occurred in all **SB-C**-doped devices, an external quantum efficiency of 10.2% was still achieved for its blue Ir(tpz)_3_ device. The deleterious effects of dopant aggregation were effectively suppressed in the **SB-Si** devices, leading to high efficiencies for green Ir(ppy)_3_ (EQE = 9.6%) and blue It(tpz)_3_ (EQE = 12.6%) devices, with negligible roll-off below 40 mA/cm^2^. Overall, the results highlight the potential of SiPh_3_-substituted spirobifluorenyl materials for use in PHOLED devices, and underscore the importance of carefully considering material morphology and intermolecular interactions in optimizing device performance.

## Figures and Tables

**Table 1:** Electrochemical data. Measurements were performed using 0.1 M TBAPF_6_ electrolyte, and the potentials are listed relative to a ferrocene internal reference.

	E_ox_^a^ (V)	E_red_^b^ (V)	HOMO ^c^ (eV)	LUMO ^c^ (eV)
**SB-C**	1.25	−3.00	−6.2	−1.3
**SB-Si**	1.34	−2.82	−6.3	−1.5

^a^ In acetonitrile; ^b^ in DMF; ^c^ calculated using the equations HOMO = −1.15 (E_ox_) − 4.79; LUMO = −1.18 (E_red_) − 4.83 according to reference [30].

**Table 2:** DSC data.

	T_m_ (°C)	T_g_ (°C)	T_sub_ (°C)
**SB-C**	284	-	245
**SB-Si**	253	87	245

**Table 3:** Summary of photophysical data.

	λ_Abs_^a^ (nm)	λ_PL_^a^ (nm)	λ_PL,77K_^a^ (nm)	λ_Exc, crystal_^b^ (nm)	λ_PL, crystal_^b^ (nm)	λ_PL, 77K crystal_^b^ (nm)	λ_Abs, film_^c^ (nm)	λ_PL, film_^c^ (nm)
**SB-C**	315	320	443	314	356	480	317	342
**SB-Si**	315	318	436	362	386	480	318	360, 430

^a^ In 2-MeTHF; ^b^ sublimed crystals; ^c^ amorphous thin film (40 nm).

**Table 4:** Summary of OLED performance.

EML	V_turn-on_^a^ (V)	EQE_max_ (%)	@ 1000 cd m^−2^		λ_max_ (nm), CIE
			EQE (%)	J (mA/cm^2^)	
9 wt% Ir(ppy)_3_					
**SB-C**	5.2	1.2	0.6	61.9	511, (0.28, 0.61)
**SB-Si**	3.9	9.6	9.6	3.2	512, (0.28, 0.63)
20 wt% Ir(tpz)_3_					
**SB-C**	2.8	10.2	3.6	11.4	488, (0.26, 0.54)
**SB-Si**	2.8	12.6	11.7	4.4	483, (0.20, 0.43)

^a^ At 1 cd/m^2^.

## Data Availability

There will be no research data provided beyond the material in the SI and available through the Cambridge Crystallographic Database.

## Supplementary Materials

The following supporting information can be downloaded at: https://www.mdpi.com/article/10.3390/molecules28135241/s1. Electrochemical data, details of molecular modeling studies and OLED fabrication/testing results.
