# Supplementary material for: A Comparison between Triphenylmethyl and Triphenylsilyl Spirobifluorenyl Hosts: Synthesis, Photophysics and Performance in Phosphorescent Organic Light-Emitting Diodes
Source: Molecules. 2023 Jul 6;28(13):5241. doi: 10.3390/molecules28135241 (PMC10343340; doi:10.3390/molecules28135241)
Supplement: Supplementary file 1 [file molecules-28-05241-s001.zip › molecules-2432401-supplementary.pdf]

Supporting Information

for

A Comparison between Triphenylmethyl and Triphenylsilyl Spirobifluorenyl Hosts: Synthesis, Photophysics and Performance in Phosphorescent Organic Light-Emitting Diodes

Wei Wei [1,†], Jie Ma [2,†], Jonas Schaab [1], Jason Brooks [3], Seogshin Kang [1], Matthew T. Whited [1], Peter I. Djurovich [1] and Mark E. Thompson [1,2,*]

[1] Department of Chemistry, University of Southern California, Los Angeles, CA 90089, USA
[2] Mork Family Department of Chemical Engineering and Materials Science, University of Southern California, Los Angeles, CA 90089, USA
[3] Universal Display Corporation, Ewing, NJ 08618, USA
* Correspondence: met@usc.edu
† These authors contributed equally to this work.

Table of Contents

Table of Contents 1

Table S1: Crystallographic Data for **SB-C** and **SB-Si** 3

Figure S1. Packing of **SB-C** (top) and **SB-Si** (bottom) molecules in a unit cell 4

Figure S2. Calculated Hirshfeld surfaces of **SB-C** and **SB-Si**, which are shown in different viewing angles (front, left and back) 5

Figure S3. DFT calculated HOMO (solid) and LUMO (mesh) surface and levels of **SB-C** and **SB-Si**. Calculated values are compared to the measured values. 6

Figure S4. (a) Cyclic voltammetry curves of **SB-Si** and **SB-C**. (b) Differential pulse voltammetry curves of **SB-Si** and **SB-C**. 7

Figure S5. Differential scanning calorimetric (DSC) thermograms of **SB-Si** and **SB-C** 8

Figure S6. Voltage dependence of EL spectra of the undoped devices. 9

Table S1: Crystallographic Data for **SB-C** and **SB-Si.**

**Identification Code**	**SB-C**	**SB-Si**
Empirical formula	$C_{44}H_{30}$	$C_{43}H_{30}Si$
Formula weight	558.68	574.76
Temperature/K	101(1)	102(2)
Crystal system	Monoclinic	monoclinic
Space group	$P2_1/n$	$P2_1/n$
a/Å	13.2736(3)	13.54470(12)
b/Å	9.03871(19)	9.29422(8)
c/Å	24.5142(6)	24.5334(2)
α/°	90	90
β/°	91.929(2)	91.1600(8)
γ/°	90	90
Volume/Å^3	2939.45(11)	3087.81(5)
Z	4	4
ρ_{calc}g/cm^3	1.262	1.236
μ/mm^1	0.541	0.888
F(000)	1176.0	1208.0
Crystal size/mm^3	0.164 × 0.11 × 0.039	0.576 × 0.201 × 0.1
Radiation	Cu Kα (λ = 1.54184)	Cu Kα (λ = 1.54184)
2Θ range for data collection/°	7.216 to 160.454	7.208 to 160.704
Index ranges	$-16 \leq h \leq 16, -11 \leq k \leq 7, -31 \leq l \leq 30$	$-17 \leq h \leq 16, -8 \leq k \leq 11, -31 \leq l \leq 31$
Reflections collected	23585	49890
Independent reflections	6180 [R_{int} = 0.0386, R_{sigma} = 0.0349]	6740 [R_{int} = 0.0498, R_{sigma} = 0.0289]
Data/restraints/parameters	6180/0/398	6740/0/398
Goodness-of-fit on F^2	1.051	1.057
Final R indexes [I>=2σ (I)]	R_1 = 0.0408, wR_2 = 0.1035	R_1 = 0.0390, wR_2 = 0.1011
Final R indexes [all data]	R_1 = 0.0489, wR_2 = 0.1078	R_1 = 0.0418, wR_2 = 0.1032
Largest diff. peak/hole/e Å^{-3}	0.30/−0.21	0.45/−0.35
**#CCDC**	**2221201**	**2216982**

SB-C Unit Cell

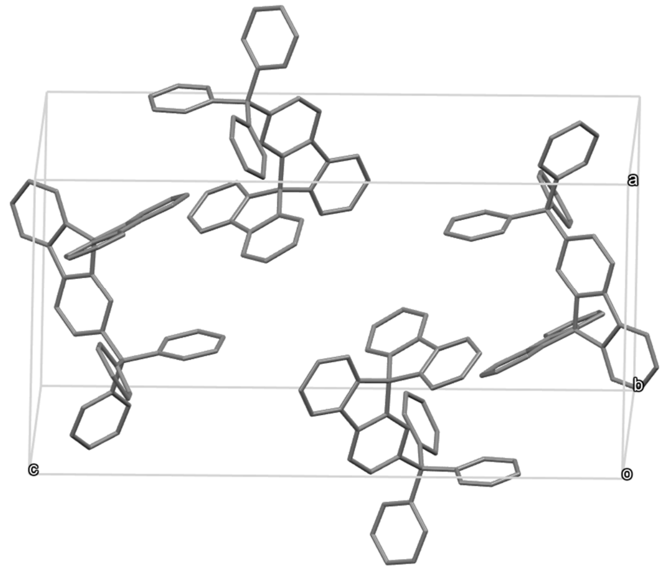

SB-Si Unit Cell

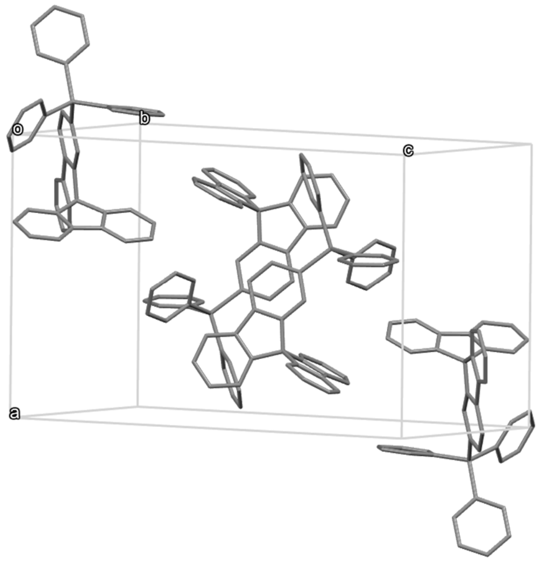

Figure S1. Packing of **SB-C** (top) and **SB-Si** (bottom) molecules in a unit cell.

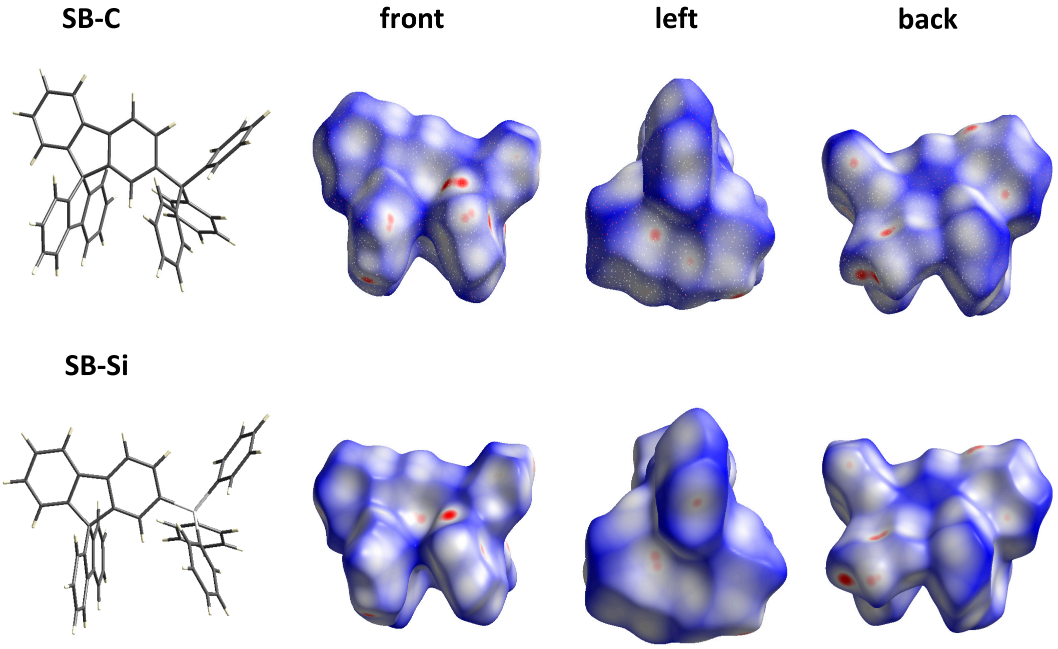

Figure S2. Calculated Hirshfeld surfaces of **SB-C** and **SB-Si**, which are shown in different viewing angles (front, left and back).

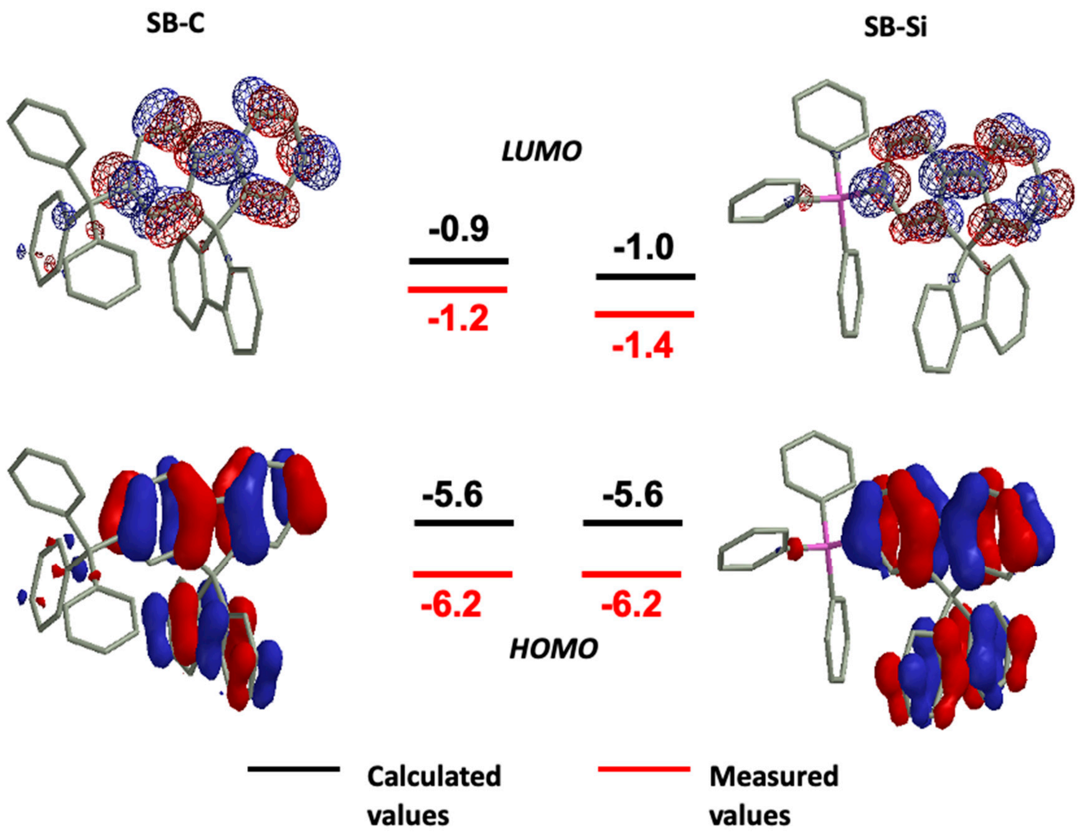

Figure S3. DFT calculated HOMO (solid) and LUMO (mesh) surface and levels of **SB-C** and **SB-Si**. Calculated values are compared to the measured values.

(a)

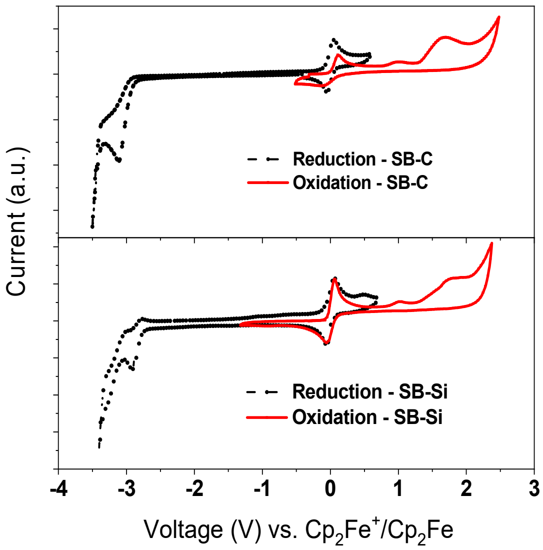

(b)

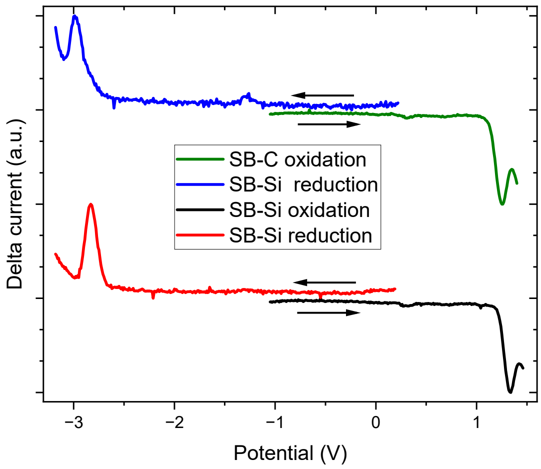

Figure S4. (**a**) Cyclic voltammetry and differential pulse voltammetry, (**b**), curves for **SB-Si** and **SB-C**.

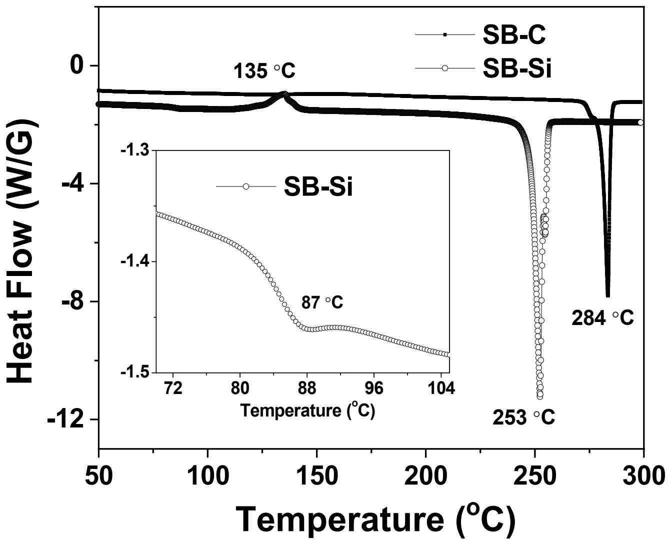

Figure S5. Differential scanning calorimetric (DSC) thermograms of **SB-Si** and **SB-C.**

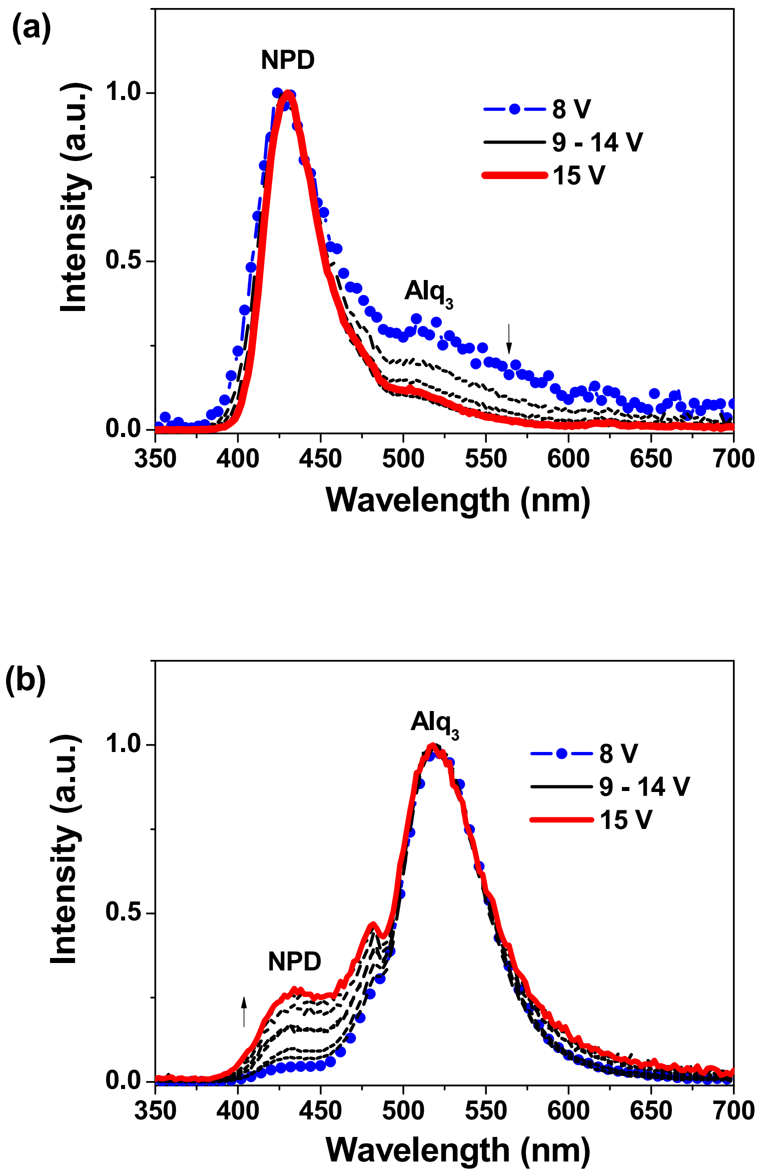

Figure S6. (**a**,**b**) Voltage dependence of EL spectra of the undoped devices.
